# Ethnicity and power in the mental health system: experiences of white British and black Caribbean people with psychosis

**DOI:** 10.1017/S2045796020001043

**Published:** 2021-02-05

**Authors:** V. Lawrence, C. McCombie, G. Nikolakopoulos, C. Morgan

**Affiliations:** Health Service and Population Research Department, Institute of Psychiatry, Psychology, and Neuroscience, King's College London, London, UK

**Keywords:** Ethnicity, social disadvantage, experiences, black Caribbean, psychiatric services, United Kingdom, qualitative

## Abstract

**Aims:**

Persistent inequalities exist in how individuals from minority ethnic groups access mental health care. A failure to investigate how these inequalities are experienced and what they mean to people with psychosis has privileged professional narratives and hindered our understanding of how they are sustained and what could be done to reduce them. The aim of this study was to investigate the long-term experience of living with psychosis and navigating mental health services within different ethnic groups.

**Method:**

Our approach was informed by work on narrative analysis and prioritised the meaning that mental health services held for participants. In-depth interviews with 17 black Caribbean, 15 white British and 3 non-British white people with psychosis as part of AESOP-10, a 10-year follow-up of an ethnically diverse cohort of individuals with first-episode psychosis in the UK. Thematic narrative analysis was used to examine experiences at the personal level within and then across the individual accounts.

**Results:**

Service users shared many defining experiences and narratives frequently returned to individuals' first contact with mental health services, first hospital admission, the experience of impatient wards, and the meaning of medication and diagnosis in their lives. We found that experiences of powerlessness punctuated the journey through mental health services and this appeared to dominate the accounts of black Caribbean, and to a lesser extent, white British participants. The findings reveal how negative expectations and experiences of mental health services are compounded over time, creating a vicious cycle of disempowerment and mistrust that manifests for many in resistance to – or at the best passive acceptance of – intervention by mental health services. High levels of need, coupled with alienation from services, contributed to negative patterns of service use among black Caribbean participants. White participants recounted substantial, though fewer, experiences of disempowerment and more instances of shared decision making that for some helped protect positive aspects of their lives.

**Conclusions:**

Against a background of entrenched social and economic disempowerment, services were experienced as disempowering by many black Caribbean people, compounding and perpetuating a sense of alienation. Concerted efforts by services to more systematically target social needs and to share power through partnership working may reduce the mistrust that many with psychosis feel when entering services and in turn reduce persistent inequalities across ethnic groups.

## Introduction

Over the past 40 years, evidence has accumulated of persistent inequalities in pathways to mental health care in the UK among minority ethnic groups, with black Caribbean people with psychosis more often accessing mental health services via the police and under compulsion than their white counterparts (Halvorsrud *et al*., 2018). In recent years, the narrow focus of this body of research has been criticised for an overreliance on a medical epidemiological framework that suggests that members of the black Caribbean population are more likely to have a coercive relationship with mental health services but provides limited insight why these adverse associations exist (Morgan *et al.*, 2004). How the pathway into care is experienced and what these established patterns mean to people with psychosis has received even less attention (Bhui *et al*., 2018). Research has described how mistrust of mental health services can contribute to a reluctance to seek help among Black and minority ethnic (BME) communities (Islam *et al*., 2015), which could create higher rates of involuntary admissions through emergency pathways. Equally, involuntary admissions may contribute to increased mistrust in this group. Research suggests a complex picture with higher levels of mistrust among people of Black ethnicity compared to White ethnicity (Henderson *et al*., 2015) directly associated with reports of ‘unfair treatment by mental health services and staff’, and related interactional issues, such as service users' not feeling heard (Rose *et al*., 2011).

In a quantitative analysis of outcome data from AESOP-10, our 10-year follow-up of an ethnically diverse cohort of individuals with first-episode psychosis (Morgan *et al*., 2017), we found that black Caribbean patients with psychosis experienced worse clinical, social, and service use outcomes than the white British majority, not only at first presentation to services (Morgan *et al*., 2008), but throughout our 10-year follow-up. Enduring social disadvantage and isolation among the black population were found to contribute to these ethnic disparities in the course and outcome of the psychotic disorder (Morgan *et al*., 2017). However, these data cannot tell us how social disadvantage and coercive pathways to care inform subsequent engagement with services and clinical outcomes. Here, we respond to calls to foreground what is at stake for the individuals involved by giving voice to the lived experiences of the black Caribbean and white British people with psychosis. In doing so, we provide detailed evidence in which to ground solutions to these complex and seemingly intractable inequalities in access and care among those with severe mental illness from ethnic minority groups (Bhui *et al*., 2018).

## Method

The aim of this study was to investigate the long-term experience of living with psychosis and navigating mental health services within the black Caribbean and white British people with psychosis. We conducted a qualitative study, embedded within AESOP-10, a 10-year follow-up of an ethnically diverse cohort on 532 individuals with first-episode psychosis in the UK, which sought to investigate patterns and determinants of the course and outcome of psychosis (Morgan *et al*., 2017).

### Data collection

Seventeen black Caribbean (BC), 15 white British (WB) and 3 non-British white (NBW) participants were purposively sampled from the AESOP-10 cohort to ensure the roughly similar number of black Caribbean and white participants and to ensure a spread in terms of gender, age, and mode of entry into services (i.e. voluntary v. compulsory) (see Table 1). Throughout the research process, attention was given to reassuring participants that all interviews were confidential, voluntary, and would not affect their care in any way. Written informed consent was obtained from all participants. Interviews were conducted by CM, a white male academic who was involved in data collection at both baseline and follow-up. Broad open-ended questions invited participants to relate their journey through mental health services in a chronological sequence, from the onset of symptoms to the present day. Interviews commenced by asking participants to tell the interviewer in his or her own word how they first came to be in contact with services. The study aimed to elicit sequential narratives, led by participants' accounts, but supplemented by prompts that encouraged participants to elaborate on motivations and meanings where necessary. Interviews typically lasted between 1 and 2 h and were conducted in participant's homes or at the research institution, according to individual preference. All interviews were audio-recorded and transcribed verbatim.

**Table 1.** Key characteristics of participants

	Black Caribbean (*n* = 17)	White British (*n* = 15)	White other (*n* = 3)
Gender:			
Male	6	9	2
Female	11	6	1
Age group:			
21–30	10	12	1
31–40	6	2	2
41–50	1	1	0
Primary diagnosis			
Schizophrenia (broad)	10	10	2
Mania	4	3	1
Depression	3	2	0
Place of birth			
UK born	13	15	1
Non-UK born	4	0	2
Economic status			
Unemployed	12	6	2
Employed	3	8	0
Economically inactive	2	1	1

### Data analysis

Our approach was informed by work on narrative analysis, specifically experience-centred research (Ricoeur, 1991; Squire, 2013) that assumes narratives are an essential part of sense making that reconstitute as well as express experience. Hence, we prioritised the meaning that mental health services held for participants. We used various strategies to raise awareness of how our own experiences and identities as researchers might influence the construction and interpretation of the research data and to guard against the further imposition of professional narratives that dominate the literature (Bhui *et al*., 2018). VL (white British female, social scientist) led the analysis, but regularly compared viewpoints on individual cases and narrative themes with CMc (white British female, former mental health practitioner and service user) and GN (Greek make, MSc student) who independently analysed transcripts and recorded interpretations in analytical diaries. CM provided ongoing input on the extent to which the analysis resonated with his experience of conducting the interviews.

Prior to analysis, VL listened to each interview, read and re-read the whole transcript and completed ‘case histories' to summarise salient patterns, plots, reflections and the dominant tone of each interview. VL then coded the interviews, using the thematic narrative analysis to examine experiences at the personal level within and then across the individual accounts (Reissman, 2008), examining commonalities and differences across participants and if and how ethnicity affected the discussion. Additional attention was given to making sense of narratives that appeared guarded, chaotic, or terse, as well as negative cases that did not appear to fit emerging trends.

## Results

The tone of narratives included critical, defiant, reflective and passive, yet it was striking that the majority sought to educate the interviewer on the experiences of powerlessness that punctuated their pathway through care and appeared to dominate the accounts of black Caribbean, and to a lesser extent, white British participants. Here we focus on five key aspects of individuals' journeys through the mental health system that reflects the typical sequencing and progression of themes and spotlight ethnic differences in the service users' experience of power and autonomy. Pseudonyms are used to protect participants' identities.

### Entering mental health services

Participants' route to mental health services, specifically the events that precipitated their first contact and the parties involved, suggested that members of different ethnic groups enter services with an unequal degree of agency and purpose. Two-thirds of white British participants described attending emergency clinics at the mental health hospital voluntarily, some having spoken with their GP first, and most accompanied by a family member or friend. Close members of their social network have been often instrumental in seeking help, providing invaluable emotional and/or practical support.

I think then Mum and Dad realised that they couldn't cope with me and that I needed specialist help.

QU: So what did they do?

*They drove me down to the Maudsley. Well they spoke to a psychologist friend of my Dad's… and he said that the best place* was *the Maudsley. (Sara, WB)*

Black Caribbean participants demonstrated a greater reluctance than white British participants to approach mental health services. Their first contact was often characterised by police involvement, itself precipitated by confrontations within the community with neighbours or family members contacting the police. Typically, individuals were then taken directly to hospital rather than to a police station, a recurring pattern that becomes commonplace for some.

*‘I think a neighbour, or somebody, must* have *called the police then they come and took me away, I said, ‘*Are *you my taxi?’ and I went in my taxi to the hospital, I knew they* were *going to take me to the hospital’ (Victoria, BC)*

Sectioning regularly followed police involvement. White British participants also recounted violent episodes prior to their first admission, but police appeared less likely to become involved. For example, both Daniel (WB) and Clare (WB) described threatening loved ones with knives, but these incidents were diffused by friends and family. Police involvement caused distress, yet it was striking that the reputation of the local mental health hospital could invoke even greater dread among black Caribbean participants and their families. Individuals cited negative personal experiences of friends or family that contributed to a fear of being put in a ‘*white jacket with the laces up*’ *(Charlie BC).* It was felt that being black exacerbated these fears.

*‘I know from, you know, my brother that works for an organisation and he, before…he worked for somebody* who dealt *with mental health and that's what they* dealt *with* was *you know people of colour being put* into *mental health, being wrongly diagnosed, totally drugged up. I* was *just, I* was *terrified’ (Everlyn, BC)*

### Admission to an inpatient unit

Discussion of individual admissions to inpatient units for the first time evoked painful memories. Participants vividly recalled their agitation and confusion and many criticised mental health professionals for failing to explain what was happening to them at a point when they were at their most vulnerable. Tessa (BC) explained that this sense of powerlessness had a lasting effect: ‘*From then I couldn't talk to* anyone, *I didn't feel I could trust* anyone *there, to be treated* like *that I just lost so* much *trust’.* Charlie (BC) reported that he was unaware that admission to hospital could be voluntary: he had been admitted twice, both times via the police, both times under a section of the Mental Health Act. He viewed the second occasion, which occurred after a two-year period of successfully managing his symptoms in the community, as unnecessary and punitive.

*It* was *my Mum, I* was *upset at her for what she had done to me, abused me, hit me you know, violently, I just didn't* like *it and I just blew up because she had hurt me, I just blew up and I took my anger out on her, you know.*

QU: And you took your anger out on you Mum just by shouting at her?

*Yeah, yeah, yeah, that's it I didn't hit her or anything… so… the police, I opened the door and they said you know ‘we've had a complaint from your Mum’, you know, ‘you've become angry and upset, you're obviously unwell, so we've got to* have *to put you in hospital again’. (Charlie, BC)*

Like many other participants, he felt that the power to decide whether he was ill resided with the mental health system, noting they *‘do whatever's necessary to make you well in their eyes*’. Many white British participants who had been admitted voluntarily viewed this as a false distinction. Though they had ostensibly given their consent, many felt coerced by family, friends, and/or health professionals to do so and that once on the ward they did not have the freedom to leave.

### Experience of impatient wards

Most reported they had encountered good and bad staff on inpatient units. Women in the sample were more likely to describe positive relationships and to praise some staff for being non-judgemental, listening and showing concern. However, other staff members were heavily criticised for treating people with mental health problems with a basic lack of humanity. Many complained that information has been rarely forthcoming.

*‘I didn't know what I* was *taking, they didn't explain to me what* was *wrong with me, then they had meetings, but they didn't* have *meetings with me involved in there, so I didn't really understand why I* was *there. But the medication, I don't know, I don't know what it* was *for.’ (Kelly, BC)*

Again, this contributed to a sense of disempowerment that could have a profound and enduring effect on participants.

*‘It* was *awful, there* was *very* few *people* who *actually cared in there, I mean to be in such an environment where people don't care, and they hold all the cards, and you* have *absolutely no rights and you* have, *you know, there's no respect, I mean it* was*…a nightmare’. (Ira, BC)*

*‘This whole experience feels* like *complete rape, a complete psychological, mental rape*’. (*Patrick, NBW)* Loss of freedom was acutely felt, and imagery of imprisonment commonplace. Restrictions were characterised as excessive and strictly regulated rules around visitors and leaving the ward made even those on voluntary admissions *‘feel* like *an animal in a cage’ (Laura, WB).* Though not directly asked, every black Caribbean woman commented upon the absence of black doctors in the health system who, it was felt, would be more likely to understand their perspective and concerns. For these participants, psychiatry appeared synonymous with ‘white’ doctors.

*‘*It's *interesting that there aren't that* many, *but yes … Africans, bring in the Africans. I'm serious, I think it would be very, very different, because unless they're completely taken in by this whole system business, which they probably wouldn't be because they* were *born somewhere else, but they'd just* have *a completely different way of hearing you when you* were *saying things’. (Paula, BC)*

The ward environment was generally considered fraught and unpleasant, yet at crucial points, it provided some with respite from the instability and social stressors that characterised their everyday lives.

*‘I don't think I did anything but lie there let my mind wander, eat, lie there, eat, but then as I got through that, it* was *horrible…because I'd got through that just needing to feel safe* kind of *period, and then I suddenly remembered what an awful place it* was*’. (Rebecca, BC)*

Many participants across the sample described chaotic lives marked by financial hardship, housing instability, emotional abuse, and violence. Black Caribbean participants were particularly likely to report family tensions arising from living together in circumstances seemingly borne out of necessity rather than choice, compounding this desire to escape from their lives. Victoria (BC) explained that she experienced cyclical inpatient admissions, which were precipitated by recurring conflict in her relationships and altercations in the community, as both a lifeline and a curse. She concluded, ‘*Yes that's my prison of course it* is*’.* Conversely, the White British families in the sample appeared to have greater social and material resources from which to draw upon to offer alternative care arrangements that simultaneously provided distance from everyday stressors and helped to avoid or curtail admissions.

*‘I went to stay with my mum in Yorkshire for I think ten days to two weeks, didn't go back to work, somebody took care of that, somebody phoned and told them, I don't know* was *told, and gradually over a two week period, probably a bit less than that, I'd come down off this highly anxious completely upside down topsy-turvy world and came back gradually more* into *reality and two weeks later I* was *back at work. I wouldn't say things* were *back to normal at that point, but I* was *certainly able to work’. (Jonathan, WB)*

*‘He [psychiatric nurse] said, “Oh he should be in hospital’, but my Mum* was *very* kind of like, *‘Oh you know I can handle it’. (Christopher, WB)*

### Positioning of medication

Participants typically depicted their first experience of medication as something that they had been done to them. Side effects of the medication were discussed at length with participants using metaphors of *‘zombie’* and *‘vegetable’* that conveyed a diminished sense of self. Participants often felt powerless, forced to take medication against their will and unable to make their concerns heard.

*‘I just felt that I wasn't being listened to even though I* was *genuinely expressing what* was *going on,* it's kind of *not being believed,* it's *what's more frustrating, especially when you're the only one that* has *to* have *the outcome of it if you take a pill or something…If I'm in a situation you know I read up on medication on the internet before I even let it pass my lips or whatever, but… they don't want that… and I've known that from, that's been my experience from day one you know.’ (Ira, BC)*

It was notable that a handful of white British participants recounted success in negotiating this aspect of their care with some arguing that a reduced dosage was necessary to continue their undergraduate degrees or careers.

*‘I knew now I* was *never going to get better, pull out this depression on 10 or 20 mg, so…. you know, I kept pushing for it to go up, and then I just said, ‘look this* is *the choice’ I said to [name of psychiatrist], I said, ‘I'm going to lose my house and my home if I don't get a job’, I said ‘you know I'm on a short-term contract, I'm sure you've been on those’, he said, ‘Oh OK Lydia’ (Lydia, WB).*

*‘I told them that I didn't think it [taking lithium]* was *a good idea. I* was *quite clear in my mind what I* was *doing, why I* was *there and what I* was *trying to do, what I* was *trying to do* was *get the Prozac out of my system, to calm down, restock and get back on with my life really’ (Oliver, WB)*

Almost all participants described episodes of reducing or stopping their medication in the community, either to escape side effects or due to a belief or hope that they could manage without it. This decision was typically taken independently of community mental health teams and presented as a rare opportunity for individuals to exert control over their illness. Val (BC) expressly stated that she took this decision herself as she felt that her doctor would dismiss her concerns. Frequently, however, this led to a worsening of symptoms and repeat hospital admissions, thereby having the perverse effect of diminishing individuals' sense of agency. A handful of participants described their despair at being subject to a seeming unending cycle of psychotic episodes, medication, non-compliance and compulsory admissions.

*‘I* was *so sick of being in and out of hospital and having to take tablets and feeling so down that I just wanted to die I think by then.’ (Daniel, WB)*

Others described a commitment to taking medication that had been established after finding the optimal dosage in partnership with community mental health professionals who many discovered were often sympathetic to the negative effects of medication. They chose to tolerate any remaining side effects as they had come to understand that medication enabled them to live their lives as fully as possible.

### Attitudes towards diagnosis

As with medication, the majority characterised receiving a diagnosis as something that was done to them by others and very few felt involved in this process. For many participants, their diagnosis represented a label that they considered to be inadequate, an oversimplification for the benefit of others. As Tessa (BC) explained, *‘I* was *immediately labelled, I didn't feel as though they took their time out to address any of my problems they* were *all just take, take, take’.* Others described tolerating the label, viewing it as a means to an end. Patrick (NBW) engaged with this process as a means to reclaim some control over his care. *‘I went to the library, I started looking up a lot of case studies and things and what I* was *reading* was *autism, right? Some similarities to autism, and schizoeffective disorder, mood changes as well, so I* was *trying to find what's the closest match and trying to relate to that, coming to terms with what* kind of *label, they're trying to put a label on me so what* kind of *label fits me best that's what I* was *looking for.’* A large proportion of women, particularly among black Caribbean participants, disputed the value of pathologising what they perceived to be an understandable reaction to acute stresses in their lives. This reflected a deep-seated and pervasive belief among participants that harmful interpersonal experiences and social circumstances were at the root of their problems. Ira (BC) asserted, *‘If the fact that outside stressful, extreme outside stressful situations and medical illness happen to me and that makes me* have, *you know, react then yes fine then, I* have *a mental illness, if that's what the definition* is*’*. Again, culture and ethnicity were deemed relevant and black Caribbean participants repeatedly criticised the narrow focus of psychiatry and its lack of engagement with other cultural constructs of mental illness.

*My critique of psychiatry* is like *I said to you before if you don't believe in the spirit world then you, or even* accept *that it exists even if you don't believe in it, then you'll* have *a hard time coping with psychiatry especially in a multi-cultural way. I mean I don't know about English people, but especially with a multi-cultural approach, a realistic one, you'd* have *a very hard time,* it's *very sterile, a lot of it* is *very sterile and it* is *very clinical, and* it's *a narrow* perspective *of life. (Paula, BC)*

## Discussion

The findings presented here provide novel insights into how negative expectations and experiences of mental health services are compounded over time, creating a vicious cycle of disempowerment and mistrust that manifests for many in resistance to – or at the best passive acceptance of – intervention by mental health services. Before considering these findings further, it is important to note several methodological issues.

### Methodological considerations

Our sample is large for a study of this sort, allowing us to investigate attitudes towards services among people with psychosis in different circumstances. However, the sample is select, and while we actively sought difference the breadth of views may not have been fully captured. Constraints on space have limited our discussion of context, though we recognise that narratives were collected by a white male academic as part of the AESOP-10 study in which illness experiences may have felt reduced to symptoms and service use. Thus, interviews may have represented an opportunity to critique the traditional clinical model of mental health. We also acknowledge that our own research interests, which coalesce around the impact of social context on mental health may have heightened our sensitivity to the limitations of the medical model in conceptualising and responding to mental distress. However, the conviction with which these insights were given, and the uniformity with which participants choose to expound the damaging impact that inequalities of power held for them, suggest that these views were deeply held.

### Mistrust and disempowerment

Our qualitative data suggests that mistrust of mental health services not only contributes to a reluctance to seek help among people of black ethnicity (Islam *et al*., 2015), but also exacerbates individuals' sense of vulnerability when services are accessed. It was evident across the sample that overt and covert restrictions on freedom and pressure to take medication contributed to inherent power differentials between service users and the mental health system, though as noted, experiences of disempowerment appeared most pronounced among black Caribbean participants. The findings are consistent with a two-dimensional view of power as an agency (Bachrach and Baratz, 1962) in which power is theorised to be behavioural and in evidence both in observable decisions (e.g. in the power of mental health professionals to enforce admission, care and treatment) and in non-decision making whereby power can be used to suppress challenges (e.g. inviting patients to enter hospital ‘voluntarily’ to avoid detainment, limiting involvement in care planning) (Breeze and Repper, 1998). Accumulatively, the high rates of admission involving the police and compulsion and the perceived failure of mental health professionals to provide information or listen to their concerns led many black service users to feel misunderstood, frustrated and imprisoned in something they did not understand. The tendency of participants to assert their right not to take the medication in the community is perhaps unsurprising in this context and is consistent with evidence that people with schizophrenia report lower levels of medication adherence if previously hospitalised against their will (Jaeger *et al*., 2013).

Yet members of all ethnic groups used metaphors of imprisonment to describe their treatment within the mental health system, echoing narratives of ‘escape’ from hospitals and unwanted treatments that have previously been found among survivors of psychosis (Thornhill *et al*., 2004). White British participants were less likely to report being formally detained under the Mental Health Act, but many felt coerced into psychiatric treatment and that others, be it family, friends, or health professionals, had motivated this decision. A growing evidence base suggests that informal coercion in the admission of voluntary mental health service users is not uncommon, but can elicit a long-lasting distrust of services (Nyttingnes *et al*., 2016).

### Structural disadvantage

Others have argued that power is a structural phenomenon, as well as a phenomenon of agency. Structural power refers to the resources that social groups collectively possess that situate them in a pre-established, unequal social relation with other groups. Here agency can be viewed as exercises of power, but this behaviour is always subject to the influence of structural power (Layder, 1985).

We know from epidemiological studies that, at first presentation to services, those from BME groups with a psychotic disorder are more likely to be socially isolated and marginalised (Morgan *et al*., 2008). This is often rooted in childhood and adult adversity, including education, employment, and housing (Morgan and Fearon, 2007). There is evidence in our data that these structural disadvantages contribute to, and are sustained by, ethnic differences in experiences of mental health services. First, family members of white British participants often acted as important advocates in their journey through services, sometimes providing care in place of admission and/or expediting discharge. It was notable that white British family members, and people with psychosis themselves, reported greater success in challenging psychiatric decisions and negotiating their treatment than their black Caribbean counterparts. There was greater evidence of shared decision making (Slade, 2017) whereby service users and clinicians worked together to adjust medication to the individual's social circumstances, making it possible for them to maintain education and careers. By contrast, black Caribbean participants often found it difficult to make their concerns heard. This may reflect several processes, including unconscious biases among staff and less access among minority ethnic groups to personal and social resources to help navigate interactions with services. One consequence of this was the resounding sense among many of the black Caribbean participants that medication and their diagnosis were imposed on them, the latter often incongruent with their own social model of their condition (see below), confirming negative expectations and exacerbating feelings of powerlessness. This, in turn, made it challenging for black Caribbean participants to maintain education and careers.

Second, however imperfect, inpatient units provided some with a refuge from the dangers and demands of everyday life that also seemed to precipitate admissions on a cyclical basis. Many participants, particularly black Caribbean, considered their problems to be a consequence of exposure over time to several negative life experiences and adversities, ranging from poor housing and inadequate income to discrimination and trauma. However, as noted, this social model often felt incongruent with clinical models and the organisation of services that prioritises diagnosis and medication. Against a background of entrenched social and economic disempowerment, services were experienced as disempowering, compounding and perpetuating a sense of alienation and mistrust that, for many, permeates all interactions with services.

### Implications

Partnership working involves listening to and engaging with patient experience, sharing information, and seeking to incorporate patient preferences within the care provided. This is one approach that may help to counter the mistrust that many black Caribbean people with psychosis and others feel when entering mental health services, thereby mitigating imbalances of power and health inequalities (Bhui *et al*., 2018). Culturally-appropriate advocacy services that are grounded in the black community and voluntary sector, and recognise the distinct needs of diverse communities, have also been proposed as a proportionate approach to help disenfranchised patients have their views heard and considered (HM Government, 2018). But this alone is unlikely to improve clinical, social, and service use outcomes in black populations or address differentials in structural power. Put simply, addressing mental health needs requires consideration of social needs, which pervade across multiple, interconnected domains of employment, income, housing, social networks and relationships, exposure to threat, violence and so on. This, tentatively, suggests developing, evaluating and implementing more systematically packages of social interventions, especially in early intervention services, utilising the collective benefit of interventions in individual domains, such as Individual Placement and Support for employment (Bond *et al*., 2013) and peer support for isolation. The prospect is that such approaches may contribute to breaking the vicious cycle of social disadvantage and alienation from services that may underpin many of the entrenched ethnic disparities in access to and experiences of mental health care that have been the focus of research for more than 40 years.

## Data Availability

In accordance with the ethical approval, full transcript data are not publicly available. This restriction was due to the re-identifiable nature of the transcripts and the importance of establishing a confidential space for participant disclosure.
